# Admissions to MD-PhD programs: how well do application metrics predict short- or long-term physician-scientist outcomes?

**DOI:** 10.1172/jci.insight.184493

**Published:** 2025-03-04

**Authors:** Lawrence F. Brass, Maurizio Tomaiuolo, Aislinn Wallace, Myles H. Akabas

**Affiliations:** 1 Department of Medicine and Pharmacology, University of Pennsylvania Perelman School of Medicine, Philadelphia, Pennsylvania, USA.; 2 Department of Pediatrics, Children’s Hospital of Philadelphia, Philadelphia, Pennsylvania, USA.; 3 Office of Biomedical Graduate Studies, University of Pennsylvania Perelman School of Medicine, Philadelphia, Pennsylvania, USA.; 4 Department of Neuroscience and Medicine, Albert Einstein College of Medicine, Bronx, New York, USA.

## Abstract

MD-PhD programs prepare physicians for research-focused careers. The challenge for admissions committees is to select from among their applicants those who will achieve this goal, becoming leaders in academic medicine and biomedical research. Although holistic practices are encouraged, the temptation remains to use metrics such as grade point average, Medical College Admission Test scores, and postbaccalaureate gap length, combined with race and ethnicity, age at college graduation, and sex to select whom to interview and admit. Here, we asked whether any of these metrics predict performance in training or career paths after graduation. Data were drawn from the National MD-PhD Program Outcomes Study with information on 4,659 alumni and 593 MD-PhD graduates of the Albert Einstein College of Medicine and the University of Pennsylvania. The Penn-Einstein dataset included admissions committee summative scores, attrition, and the number and impact of PhD publications. Output metrics included time to degree, eventual employment in workplaces consistent with MD-PhD training goals, and self-reported research effort. Data were analyzed using machine learning and multivariate linear regression. The results show that none of the applicant metrics, individually or collectively, predicted in-program performance, future research effort, or eventual workplace choices even when comparisons were limited to those in the top and bottom quintiles.

## Abstract 2

Quantifiable metrics of MD-PhD program applicants, including GPA, MCAT score, and postbaccalaureate gap length, do not predict outcomes consistent with the goals of MD-PhD programs.

## Graphical abstract

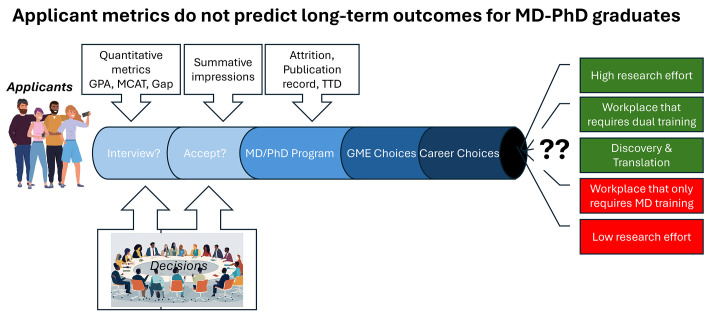

## Introduction

Physician-scientists serve a critical role at the interface between biomedical research and patient care (1–3). Over the past 6 decades, training in MD-PhD programs has become a major, but by no means the only, path to a career as a research-active physician-scientist (4, 5). Though MD-PhD program alumni constitute only 3% of medical school graduates, over half of the National Institutes of Health (NIH) research project grants held by physicians are held by MD-PhDs (4, 6). NIH National Institute of General Medical Sciences (NIGMS) Medical Scientist Training Program (MSTP) training grants support 53 MD-PhD programs, and outcomes studies show that most graduates pursue careers consistent with the program’s training mission (7–10). Thus, one might conclude that the MD-PhD admissions process is working well. However, not every MD-PhD program matriculant graduates, not every graduate has a research-focused career, and programs and their alumni are often less diverse than the applicant pool and far less diverse than the US population (7, 9, 11–17). To date, few studies have determined whether quantifiable applicant metrics predict metrics of short- and long-term success.

For most MD-PhD programs, the program organizes the admissions process in coordination with the MD admissions committee. The committee’s goal is to identify a diverse group of applicants who will excel in the medical and research phases of the program, complete the program with minimal attrition, and most importantly, pursue careers as research-focused physicians. Each year over the past decade, approximately 1,800 individuals applied to MD-PhD programs and 700 matriculated (16, 18). Individual programs receive 20 to 900 applications and interview 10 to 120 applicants for entering classes that range from 1 to 30 or more (19). MD-PhD admissions committees are the de facto gatekeepers who determine who will be allowed to enter the training pathway and therefore who will constitute a substantial fraction of the future physician-scientist workforce.

### The basis for admissions decisions.

The MD-PhD program admissions process has 2 critical decision points: deciding whom to interview and whom to accept. The initial screening process relies on information in the American Medical College Application Service (AMCAS) application and school-specific secondary applications. Applicant information can be divided into 2 categories. The first includes quantifiable metrics such as undergraduate grade point average (GPA), Medical College Admission Test (MCAT) scores, length of the postbaccalaureate gap between college and medical school (20), publications, presentations (posters and talks), and sociodemographic status including family socioeconomic status (SES) and parental education/income (21). The second category includes nonquantifiable characteristics gleaned from application essays, activities, and letters of recommendation. These include (a) passion for science, research, and discovery; (b) intellectual curiosity; (c) creativity; (d) perseverance; (e) resilience; (f) grit; (g) maturity; (h) work ethic; (i) capacity for empathy, compassion, and warmth; and (j) communication skills. A recent Supreme Court of the United States decision has constrained the use of race and ethnicity in this process.

In 2022, the average applicant GPA and total MCAT score were 3.68 and 511, respectively. The matriculant averages were higher: 3.82 and 516 (22). Public perceptions of what admissions committees are looking for can affect applicant behaviors. For example, admissions committees may consider postbaccalaureate gap length as a surrogate for commitment to a research-active career. Partly as a result, most applicants now take a gap and most devote the time to full-time research jobs (20). However, whether gap length is a good surrogate is uncertain.

*How good are the metrics as predictors?* Here, we ask whether quantifiable applicant metrics, alone or in combination, predict outcomes consistent with the goals of MD-PhD training programs both in-program and after completion of postgraduate training. We also examine whether the number and impact factor of publications during the PhD phase predict favorable workplace after completion of postgraduate training.

**Figure 1:**
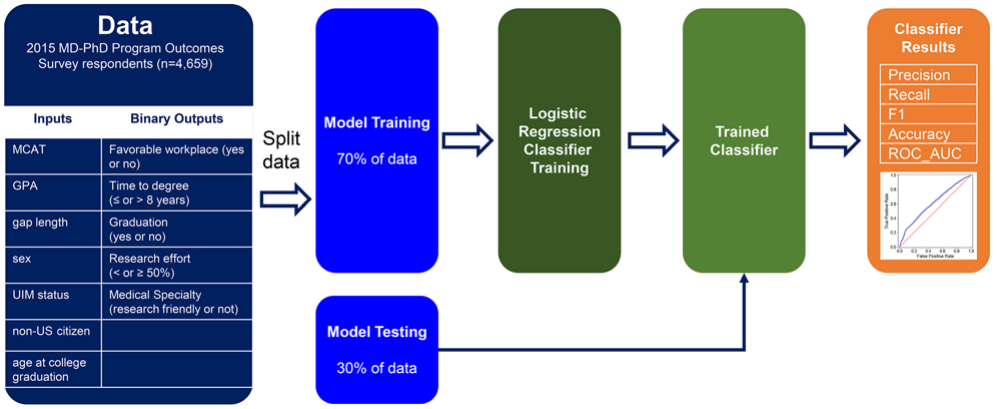
Machine learning analysis flowchart. The national dataset contains input metrics and binary outputs for 4,659 respondents to the 2015 National MD-PhD Program Outcomes Survey. The data were split, with 70% used to train the Logistic Regression Classifier and 30% used for model testing in the Trained Classifier. The Classifier results are precision, recall, F1, accuracy, and receiver operating characteristic area under the curve (ROC_AUC). A value of 0.70 for the ROC_AUC was used as the lower threshold of acceptable discrimination (49). UIM, underrepresented in medicine.

Two complementary datasets were analyzed. The first includes data on 4,659 alumni of 80 MD-PhD programs who had completed postgraduate training and completed a survey in 2015 as part of the National MD-PhD Program Outcomes Study (7–9). For the present study, the Association of American Medical Colleges (AAMC) supplemented the deidentified survey responses with the final MCAT score and GPA reported in the AMCAS medical school application system, and demographic data from the AAMC database. This is referred to as the national dataset. The respondents characterized their current position (academia, private practice, pharma/biotech industry, etc.); percentage professional effort devoted to research, clinical care, teaching, and administration; and medical specialty. We used machine learning to determine whether quantifiable metrics available at the time of application to MD-PhD programs, alone or in combination, predicted success in the programs or after completion of postgraduate training (Figure 1). Since the national dataset does not contain information about admissions committee rankings, attrition, or publication records, we also queried a second, smaller dataset that includes this information for individuals who had joined the MSTPs at the University of Pennsylvania Perelman School of Medicine and the Albert Einstein College of Medicine, referred to as the Penn-Einstein dataset. The primary conclusion is that within the range of metrics for individuals graduating from MD-PhD programs, the quantifiable applicant metrics fail to predict successful outcomes either during the program or after completion of postgraduate training.

## Results

### Classification of outcome metrics.

Except where noted, we used machine learning rather than traditional multivariate regression to analyze the data because we sought to determine, at the level of the individual, whether a combination of the variables describing an applicant could be used for outcome prediction. Outcome metrics were classified as favorable or unfavorable (Table 1). The average time to degree (TTD) for MD-PhD program graduates is currently 8.3 years (8). For this analysis, TTD is an integer: the year of graduation minus the year of matriculation. A favorable TTD was defined as less than or equal to 8 years based on the goal stated in the NIGMS MSTP T32 program announcement, PAR-24-128 (23), that training should be shorter than if both degrees were obtained sequentially (24). Workplaces classified as consistent with MD-PhD training goals (i.e., favorable) include academia, federal/government agencies, the NIH, a nonfederal research institute, and pharmaceutical and biotech industries. Workplaces classified as being inconsistent with MD-PhD training goals (i.e., unfavorable) included consulting/law/finance, private practice, and other (8). Physician-scientist–friendly graduate medical education (GME) choices were defined as those for which at least 35% of the National MD-PhD Outcomes survey respondents in that specialty reported at least 50% research effort (8). By this definition, physician-scientist–friendly choices included internal medicine, medical genetics, neurology, pathology, pediatric neurology, pediatrics, and psychiatry. The less friendly choices included all other GME specialties. For the Penn-Einstein dataset an additional outcome metric was attrition: i.e., withdrawing from the program without one or both degrees, irrespective of the reason(s). Supplemental Table 1 (supplemental material available online with this article; https://doi.org/10.1172/jci.insight.184493DS1) summarizes the comparisons that were made, the methods of analysis, and which dataset was analyzed for a comparison.

### Data distributions for applicant metrics.

**Figure 2:**
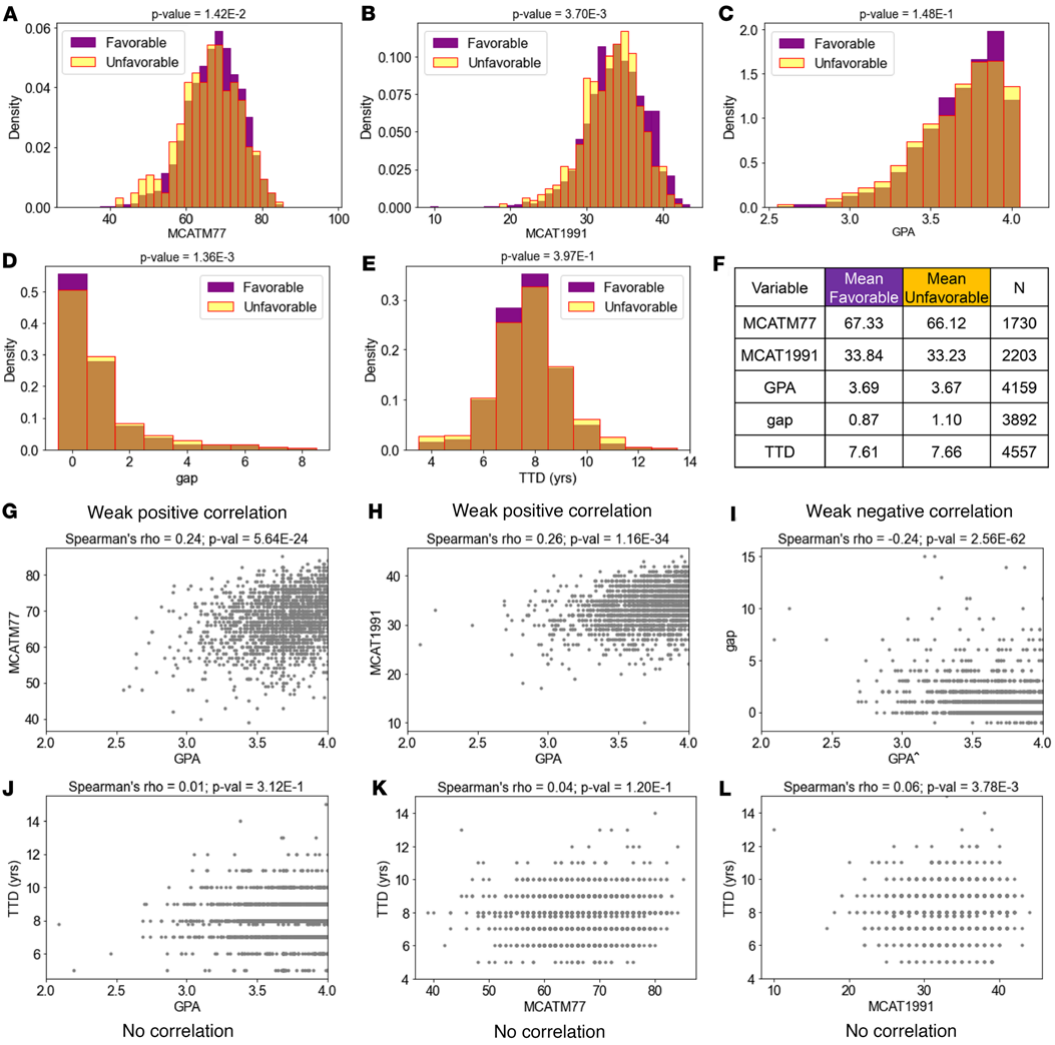
Distributions of input variables for the national dataset population based on favorable or unfavorable current workplace (Table 1) and possible correlations between pairs of input variables from the national dataset. (**A**–**E**) Histograms of input variable for favorable workplace (purple), unfavorable workplace (yellow), and the overlap (brown). *P* value above each panel indicates the significance of the difference between favorable or unfavorable distributions. Distributions of (**A**) MCATM77 scores, (**B**) MCAT1991 scores, (**C**) undergraduate GPA, (**D**) length of gap between undergraduate college graduation year and MD-PhD matriculation year, and (**E**) TTD calculated by year of MD-PhD graduation minus year of matriculation. (**F**) Mean of favorable and unfavorable groups and number of individuals for each variable. (**G**–**L**) Scatterplots for pairs of variables. The Spearman’s rho parameter is shown above each panel. For a perfect monotonic relationship between 2 variables, the Spearman’s rho coefficient would be 1 or –1 for a positive or negative correlation, respectively. A Spearman’s rho coefficient close to 0 indicates no correlation. The panels show scatterplots between data pairs (**G**) GPA and MCATM77, (**H**) GPA and MCAT1991, (**I**) GPA and gap length in years, (**J**) GPA and TTD, (**K**) MCATM77 and TTD, and (**L**) MCAT1991 and TTD. Because MCAT scores from different versions of the exam, MCATM77 and MCAT1991, have different content and the scores are not directly comparable, we analyzed them separately. The maximum number of points in each plot is shown in **F**.

Figure 2, A–E, shows histograms of MCAT 1977, MCAT 1991, undergraduate GPA (range 2.09–4.00), postbaccalaureate gap length, and TTD for those who reported a favorable versus unfavorable workplace in the national dataset. The differences in the means for the MCAT scores and gap length for the 2 outcome groups were significantly different, in part because of the large *N* (Figure 2, A, B, D, and F). However, the extensive overlap of the distributions shows that at the individual applicant level they are not predictive of whether the current workplace was classified as favorable or unfavorable. The means for GPA and TTD were not significantly different between the 2 outcome groups (Figure 2, C, E, and F).

Supplemental Figures 1–3 show the distribution of applicant metrics and TTD for men versus women (Supplemental Figure 1), groups UIM versus non-UIM (Supplemental Figure 2), and individuals who reported a high (80%–100%) versus low (0%–20%) research effort after completion of postgraduate training (Supplemental Figure 3). While some of the differences in the means are significant, it is important to note that there is a high degree of overlap. Also, gap length and TTD have increased substantially over the past 5 decades, and the proportion of women and UIM graduates was much lower in the period prior to 2000 (7, 9).

### Possible correlations between GPA, MCAT scores, gap length, and TTD.

We used Spearman’s correlation to investigate whether correlations exist between pairs of input metrics (Figure 2, G–L). A Spearman’s rho coefficient of 1 or –1 would indicate a close correlation between the 2 variables. There were weak positive correlations between GPA and both the 1977 (Spearman’s rho = 0.25) and 1991 (Spearman’s rho = 0.26) versions of the MCAT; i.e., people with higher GPA tended to have higher MCAT scores on both tests (Figure 2, G and H). There was a weak negative correlation between GPA and gap length (Spearman’s rho = –0.24); i.e., people with higher GPA tended to have shorter gaps (Figure 2I). No correlation existed between either GPA (Figure 2J) or MCAT (Figure 2, K and L) and TTD. This means that neither GPA nor MCAT score individually predicts TTD in the program.

### Quantifiable applicant metrics do not predict a shorter TTD.

**Figure 3:**
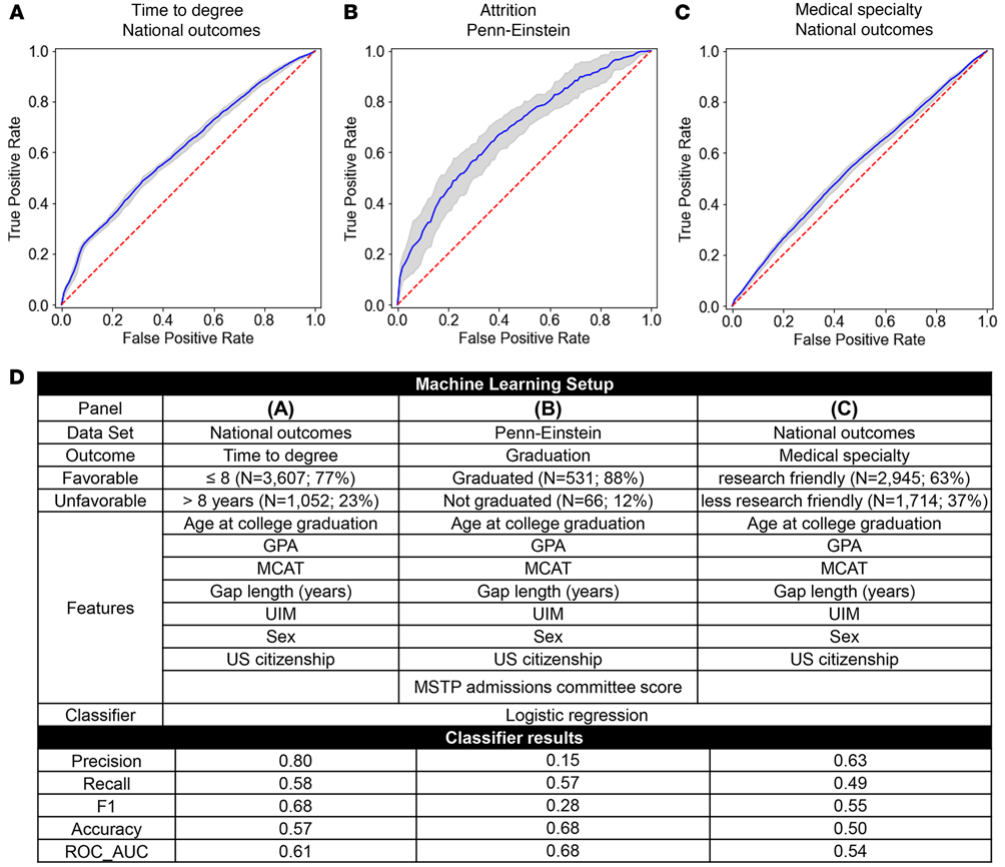
Applicant metrics do not predict in-program outcomes including TTD, attrition from MD-PhD programs, or medical specialty choice. (**A**–**C**) ROC curves for machine learning analysis of the relationship between the input metrics listed under Features in panel **D**. The ROC_AUC would be 0.0 if the model predictions were completely wrong, 1.0 if the predictions were completely correct, and 0.5 if the predictions were random (red dashed line). The blue line shows the calculated curve with the gray lines showing the 95% confidence interval for the repeated trials of the model. Favorable and unfavorable outcomes are defined in Table 1 and **D**. (**A**) ROC curve for prediction of TTD using national dataset applicant metrics (Features). (**B**) ROC curve for the prediction of graduation versus attrition outcome using the Penn-Einstein dataset. (**C**) ROC curve for the prediction of research-friendly medical specialty choice using the national dataset applicant metrics. (**D**) The dataset used, the favorable versus unfavorable outcome metric, input features (i.e., applicant metrics), and classifier results for **A**–**C**. Classifier results show the values of 5 commonly used prediction metrics. A value of 1 for each metric would indicate all correct predictions. General guidelines suggest that a value of 0.70 for the ROC_AUC is the lower threshold for acceptable discrimination (49).

TTD is an important outcome metric for evaluation of MSTP training grant applications (23). For this study we defined a favorable time to degree as ≤8 years and a less favorable TTD as >8 years. Since individual metrics did not predict a favorable versus unfavorable TTD, we asked whether a machine learning analysis based on all the applicant metrics combined (age at college graduation, GPA, MCAT score, gap length, membership in a group UIM, US citizenship, and sex) could do better. As shown in Figure 3, A and D, it did not.

Since the average TTD in MD-PhD programs has increased over the past 5 decades (9), we asked whether this increase might account for our inability to predict TTD from applicant metrics. To do this, we limited the analysis to alumni who graduated in 1995 or after (*N* = 3,041), when TTD already exceeded 8 years, and asked whether this would improve the prediction of TTD from applicant metrics. It did not (Supplemental Figure 4A).

### Augmented Penn-Einstein dataset applicant metrics do not predict attrition.

Trainee retention is an important MSTP training grant evaluation metric (23). Analysis of AAMC data for students entering MD-PhD programs between 2004 and 2012 indicates that average program attrition was approximately 16%, similar to an earlier 24-program study (25, 26). Since the National MD-PhD Outcomes alumni survey did not include matriculants who did not complete the program, we used the smaller Penn-Einstein dataset to determine whether applicant metrics could predict attrition at the individual level. We defined a favorable outcome as graduation with both degrees (*N* = 523). An unfavorable outcome was defined as withdrawal without one or both doctorates (*N* = 70). As input metrics we used GPA, MCAT score, gap length, UIM status, US citizenship, sex, age at undergraduate graduation, and the MSTP admissions committee summative scores for individual applicants. Collectively, these metrics did not predict which students left the program (Figure 3B).

### Applicant metrics do not predict the number or impact factor of PhD thesis publications.

One measure of PhD thesis research productivity is the total number of papers and the number of first-author papers published. The distributions for the Penn-Einstein dataset for number of first-author and total number of publications resulting from the PhD were similar, though they were statistically different (Supplemental Figure 5, A and B). The average number of PhD thesis papers per student was 5.4, and the average number of first-author papers per student was 2.2. The average and total impact factor of the PhD publications for the Penn alumni were shifted to higher values compared with the Einstein alumni (Supplemental Figure 5, C and D). The distributions of GPA and MCAT scores for Penn and Einstein were also markedly different. The mean GPA was 3.85 at Penn and 3.68 at Einstein. The mean MCAT was 36.5 at Penn and 31.5 at Einstein (Supplemental Figure 6).

We used a multivariable linear regression analysis to determine whether the applicant metrics could be used to predict the average impact factor for the students’ PhD thesis publications, the total impact factor of their thesis publications, or the number of publications. For this analysis, we chose a random subset of 70% of the alumni as the training set and predicted the average and total impact factor for the remaining 30%. The predicted and actual average and total impact factor, and number of PhD publications for the remaining 30% of alumni, are shown in Supplemental Figure 7. For a perfect prediction, all the data points would lie along the diagonal. We repeated the random selection of the training set multiple times with the same conclusion: The applicant metrics do not predict either the number or the average or total impact factor for the PhD thesis publications.

### Applicant metrics do not predict GME clinical specialty choice.

Our previous analysis of the national dataset showed that choice of medical specialty strongly influences the likelihood that a graduate had a position in academia or devoted at least 50% effort to research (8). More than one-third of alumni in internal medicine, medical genetics, neurology, pathology, pediatric neurology, pediatrics, and psychiatry reported at least 50% research effort. For this analysis, we defined these specialties as a favorable outcome and all others as less favorable (Table 1). Sixty-three percent of the alumni (2,945 of 4,659) trained in one of the favorable specialties. The machine learning analysis, using TTD and the applicant metrics, failed to predict who would choose a favorable versus less favorable specialty (Figure 3C).

Choices of clinical specialty by MD-PhD program graduates have changed over the 5 decades of alumni in the national dataset (7). We asked whether this change was responsible for our inability to predict which students would choose research-friendly specialties by limiting the analysis to trainees who graduated after 1994 (*N* = 3,041), a period when specialty choices were relatively stable. The results were the same. There was no relationship between application metrics (plus TTD) and specialty choice (Supplemental Figure 4B).

### Applicant metrics do not predict favorable workplace outcomes.

**Figure 4:**
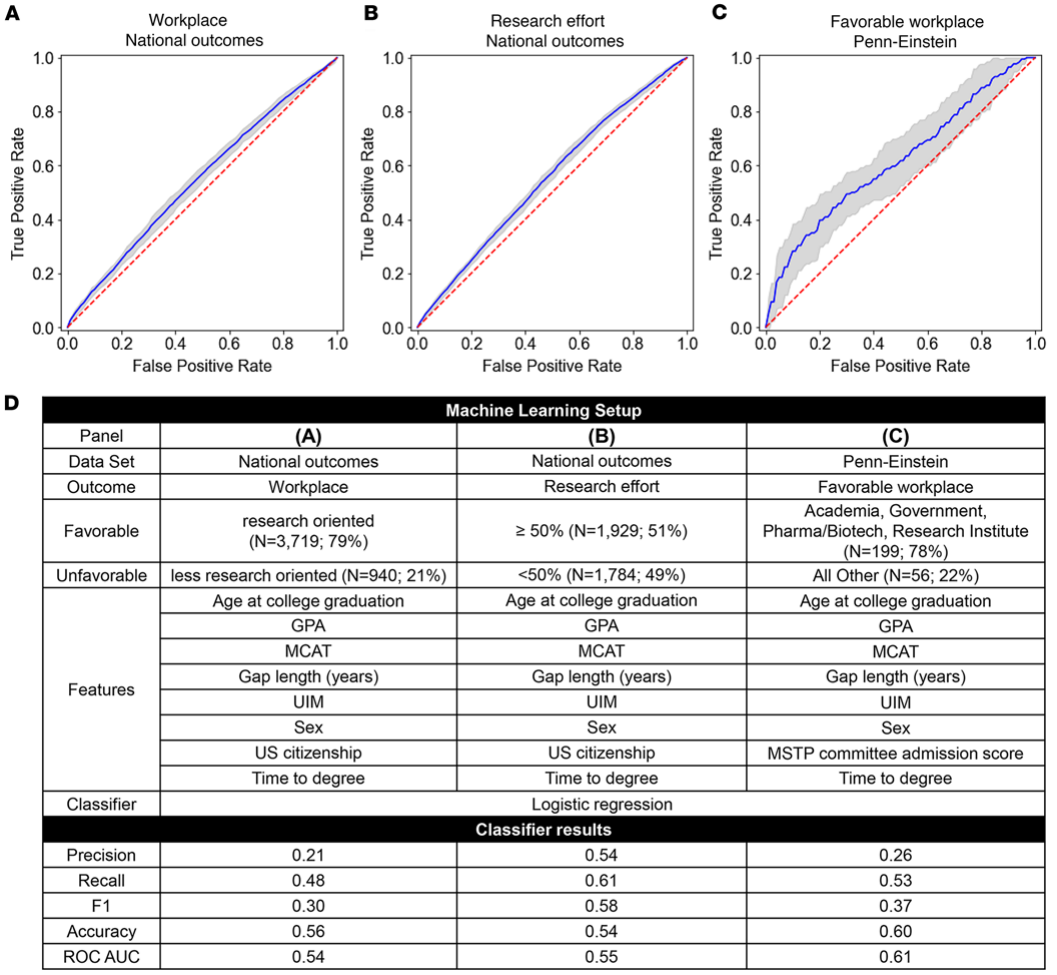
National dataset applicant metrics do not predict outcomes after completion of postgraduate training including favorable workplace or self-reported research effort for graduates in favorable workplaces, and the more extensive applicant metrics for the Penn-Einstein dataset, including publications during PhD, do not predict favorable workplace following completion of postgraduate training. (**A**–**C**) ROC curves for machine learning analysis prediction of indicated outcome based on the input metrics listed under Features in **D**. Colors and interpretation are as in Figure 3’s legend. (**A**) ROC curve for prediction of the eventual choice of a research-oriented workplace after completion of postgraduate training using the national dataset. (**B**) ROC curve for prediction of research effort classified as favorable (≥50%) or unfavorable (<50%) for graduates in favorable workplaces. (**C**) ROC curve for prediction of favorable workplace from Penn-Einstein dataset. (**D**) The dataset used, the favorable versus unfavorable outcome metric (Table 1), input features (i.e., applicant metrics), and classifier results for **A**–**C**.

The goal of MD-PhD programs is to train research-active physician-scientists. National MD-PhD Outcomes Survey respondents categorized their current workplace using a predefined list of terms. We defined favorable workplace outcomes to include academia, the NIH, a federal agency other than the NIH (e.g., the CDC and FDA), a research institute, or industry (biotech or pharmaceutical) and an unfavorable outcome as private practice, consulting/law/finance, or other (Table 1). The inputs included the available applicant metrics and TTD. As shown in Figure 4, A and D, the analysis did not predict a favorable versus unfavorable workplace outcome.

We also tested whether the ability to predict the choice of favorable versus unfavorable workplaces would improve if we limited the analysis to individuals with the highest and lowest quintiles of GPA or MCAT scores. It did not (Supplemental Figure 8, A, B, and D). Limiting the analysis to individuals with the highest and lowest reported research effort quintiles also did not improve workplace prediction (Supplemental Figure 8C).

### Applicant metrics do not predict research effort for those in favorable workplaces.

For alumni in favorable workplaces (*N* = 3,713), we tested whether the applicant metrics could predict favorable research effort, defined as at least 50% (Table 1). The machine learning analysis was unable to predict favorable research effort (Figure 4B). We also examined whether limiting the national dataset to the top versus bottom quintiles of GPA or MCAT scores improved the ability to predict research effort. It did not (Supplemental Figure 9, A–C).

### For the Penn-Einstein dataset, adding PhD publication metrics to other input metrics does not improve prediction of favorable workplace.

Unlike the national dataset, the Penn-Einstein dataset included all publications resulting from PhD thesis research. We examined whether including the publication metrics, total number of publications, number of first-author publications, and average and total impact factor of the thesis publications would improve the ability to predict favorable workplace outcomes after completion of postgraduate training (*N* = 255). Using the publication metrics in addition to applicant and other in-program metrics, the machine learning algorithm was unable to predict the favorable outcome (Figure 4C).

## Discussion

The MSTP program announcement (PA-24-128) sets MD-PhD training goals as developing “…a diverse pool of well-trained clinician scientists, who have the…knowledge, professional skills and experiences required to identify and transition into careers in the biomedical research workforce that utilize the dual-degrees…” (23). Despite the lack of evidence to support the practice, the applicant screening process tends to rely on quantifiable metrics because it is easier to compare applicants. In addition, some of these metrics determine national medical school rankings and, to some extent, success in the MD training portion (22, 27–32). Admissions committees tend to be risk averse with applicants with lower MCATs and GPAs. However, to date, the most critical question of all has not been answered: Do quantifiable metrics, alone or in combination, predict which applicants will become research-focused physician-scientists and leaders in academic medicine and biomedical research? To answer this question, 2 datasets were analyzed: a large, national dataset and a smaller, Penn-Einstein dataset that includes the same information plus 3 additional metrics: the admissions committee summative ranking that incorporates a holistic review of applications plus interview performance, attrition data, and the graduate school publication record. Even with these enhancements, the data in this second set were unable to predict performance within the program, attrition from the program, or a favorable current workplace. Similar conclusions have been reached previously in single-institution studies, including one from the MD-PhD program at Vanderbilt University (33).

Greater than 75% of entering MD students come from families in the top 2 income quintiles. This has been shown to bring them benefits with respect to MCAT preparation, career exposure, and research opportunities (34). However, it also raises an important question. In an era when greater diversity within the physician-scientist workforce has become a national goal, does reliance on quantifiable applicant metrics in the screening process and admissions decisions create a systemic bias in favor of applicants from privileged backgrounds?

### Limitations.

Before considering the implications of these findings further, it is worth considering their limitations. First, this is a retrospective, not a prospective, study, and all the study participants had been admitted to MD-PhD programs. Thus, it is uncertain whether the results can be generalized prospectively to future applicants, even those whose applicant metrics fall within the ranges analyzed here. It should also be noted that the versions of the MCAT exam (MCATM77 and MCAT1991) taken by the alumni in this study are not directly comparable with the current MCAT exam. As the AAMC states: “The MCAT exam tests the foundational concepts and reasoning skills students need to be ready for today’s medical school. Scores… help admissions officers and their committees make informed decisions about applicants’ academic readiness for the medical school curriculum.” (35). In other words, the test is not designed to predict long-term outcomes. Does including qualitative, holistic impressions help? It would be attractive to think so, but when we included summative admissions committee scores in the Penn-Einstein dataset, we were still unable to predict attrition or long-term outcomes.

### Implications.

These results suggest that desirable and less desirable outcomes cannot be predicted by applicant GPA, MCAT scores, and gap length over a wide score range, either alone or in combination. Given that, what might be the impact of using these scores to evaluate which applicants to interview or admit? These metrics have been shown to correlate with family SES, parental and family educational attainment, and race and ethnicity, as does the SAT exam score (36, 37). Grade inflation at highly selective private universities is greater than at public universities (38). Individuals from low-SES families, first-generation college students, and those from groups UIM are more likely to attend public universities (36, 39). This suggests that using GPA as a predictive metric for application screening may bias decisions of whom to interview toward those from privileged socioeconomic and educational backgrounds. Perhaps in recognition of these issues, the most recent MSTP program announcement (PA-24-128) dropped GPA reporting and states, “The [admissions] process should consider, consistent with applicable law, metrics beyond undergraduate organization prestige, grade point average, and standardized test scores.”

Another factor that may encourage admissions committees to interview applicants with the highest GPAs is the US News & World Report rankings. Many schools seek to achieve the highest possible ranking, and this may lead to an overweighting of GPA and MCAT scores in application screening and bias against applicants from diverse backgrounds (40). Recently, a number of medical schools have refused to participate in the US News & World Report medical school ranking data collection (41). Perhaps all schools should follow suit to end a ranking system that creates a systemic bias in favor of applicants from high-SES families. In addition, medical school admissions committees tend to be “risk averse.” Thus, they tend to choose to interview applicants with high GPA/MCAT scores despite limited evidence that this screens for individuals who will be the best physicians.

Similar to GPA, MCAT scores are correlated with family SES, parental educational attainment, and race and ethnicity (42, 43). Medical students from lower SES families tend to have lower MCAT scores (29). Of note, medical school applicants in the middle third of MCAT scores are more diverse than those in the upper third in terms of family SES and race and ethnicity (32). MCAT scores do correlate with successful completion of medical school. However, the effect sizes range from small to medium over a wide range of MCAT scores (28, 30, 31, 44–47). AAMC data indicate that 93% of students with a GPA of 2.80–2.99 and MCAT score of 502–505 will successfully graduate medical school in 5 years, which is not terribly different from students with a GPA of 3.80–4.00 and MCAT of 518–528, 97% of whom graduate medical school in 5 years, per Table 8 in ref. 47. Medical students from lower SES families scored lower on the MCAT but did as well or better on the USMLE Step 2CK exam than students from higher SES family backgrounds (29). Thus, the MCAT behaves more like a permissive exam. Above a score of about 502, the probability of successful completion of medical school in 5 years does not change much. Given the correlation between MCAT scores and family SES, reliance on MCAT scores in the applicant screening and admissions process merely favors applicants from higher SES families.

The third quantifiable metric available for MD-PhD program applicants is gap length between college graduation and matriculation. Over the past decade the percentage of matriculating students who have taken a postbaccalaureate gap of 1 or more years has increased from about 50% to more than 75% (20). In a recent survey, nearly all the students who reported taking a gap spent it working in research settings, yet that additional research experience did not reduce TTD (20). Program directors reported that gaps were a factor in interview and admissions decisions. Over 70% said they were definitely or probably predictive of the likelihood of completing the MD-PhD program, and 57% thought they were definitely or probably predictive of long-term commitment to a physician-scientist career (20). At odds with these impressions, the analysis here suggests that gap length is not predictive of either attrition or long-term commitment to a workplace consistent with the goals of MD-PhD training. Thus, neither taking a gap nor gap duration has predictive value for outcomes that are the essence of physician-scientist training. Given that taking a gap does not improve TTD (20), and does not predict attrition or long-term outcome, we wonder why the percentage of applicants taking a gap has increased over the past decade. Presumably this is, at least in part, because applicants perceive that it is important to gain admission.

*Does including overall impressions improve the ability to predict who will do well?* The admissions committee scores represent a holistic evaluation of applicants based on their AMCAS and secondary applications and their interview evaluations. It is noteworthy that in the Penn-Einstein dataset, attrition from the programs could not be predicted based on applicant metrics and that inclusion of the admissions committee scores did not improve prediction of attrition. This suggests that the events that lead to attrition are not predictable at the time of application/interview. Race and ethnicity were not a predictor of attrition in the analysis of the Penn-Einstein dataset. A recent study found that Black or African American trainees who matriculated between 2004 and 2012 had a higher attrition rate from MD-PhD programs compared with Hispanic and non-UIM trainees (26). The reasons for this difference are uncertain. Given that attrition from MD-PhD programs is low, averaging in the 10%–15% range (25), the admissions selection process effectively identifies individuals committed to the training pathway. Those who leave the program do not appear to be predictable based on information available at the time of application, at least not the types of information included in the present study, which includes the interviewers’ assessment of commitment.

Including summative scores also failed to improve predictions of other important outcomes, including TTD, the number of PhD publications, and the average or total impact factor of the publications. Perhaps the score range for the accepted applicants was too narrow. These outcome metrics are probably more a function of mentor and thesis research project choice and factors that are not reflected in quantifiable applicant metrics, such as GPA or MCAT scores.

Disappointingly, inclusion of in-program productivity metrics, such as TTD, numbers of publications, and average or total impact factor of the publications, also did not allow prediction of favorable career workplace outcomes. It is possible that the Penn-Einstein dataset of 255 graduates who have completed training is not sufficiently large, but it does raise the question as to whether extending training duration to achieve a paper in a high–impact factor journal is in the trainees’ best long-term interests. Over 80% of internal medicine Physician Scientist Training Program directors indicated that having a first-author paper was very important, but only 10% thought that it was very important to be in a high-impact journal (48).

### Conclusions.

Taken together, this analysis suggests that (a) over a broad range of GPA, MCAT scores, and gap lengths, quantifiable applicant metrics, alone or in combination, do not predict outcomes consistent with the goals of MD-PhD training either in program or following completion of postgraduate training, and (b) adding in admissions committee scores, TTD, number of total and first author publications, and average and total impact factor of publications does not improve predictive value. The time to complete MD-PhD programs has risen from 6.7 years for the cohort that graduated 1975–1984 to 8.3 years for the cohort that graduated 2005–2014 (8). While there are probably many causes for this increase, one is almost certainly the pressure to publish multiple papers in high-impact journals. High-impact papers are almost certainly helpful to the mentor’s pursuit of future grant support. However, the analysis here does not support the hypothesis that trainees with high-impact papers are more likely to pursue goals consistent with MD-PhD training. This raises the question of whether the increased training duration and the pressure to publish high-impact papers are prolonging TTD and not producing better graduates.

Finally, the success of trainees with GPA and MCAT scores well below the average raises the question of whether using GPA and MCAT scores to screen applicants introduces a systemic bias against applicants from lower SES families and diverse backgrounds. We should use metrics that have predictive value, not simply to achieve the highest average matriculant scores for comparisons with other programs on NIH training grants and in national rankings like the US News & World Reports. Applicants from low-SES families and first-generation college graduates are more likely to apply with lower scores and less likely to be among those who matriculate into MD-PhD programs (15). We close by encouraging programs to reexamine how these metrics are used in their admissions process and to examine the impact of this use on the diversity of the trainees that they choose to interview and accept. This may go a long way toward increasing the diversity of the next generation of physician-scientists without compromising the quality of the trainees and graduates.

## Methods

See Supplemental Methods for more extensive descriptions of the data sources and handling of imputed data and for missing values (Supplemental Table 2).

### Data sources.

Two datasets were used. One dataset was derived from the 2015 National MD-PhD Program Outcomes Study and is referred to as the national dataset (7–10). It included survey data from 4,659 MD-PhD program graduates who matriculated between 1965 and 2007 and who had finished postgraduate training when they completed the survey in 2015 and their final MCAT score and undergraduate GPA. The national dataset machine learning analysis was divided into 2 endpoints, prediction of in-program and post-program outcomes. For in-program outcomes the input features used were applicant metrics that included final MCAT score, undergraduate GPA reported in the AMCAS application, gap length (number of years between college graduation and MD-PhD program matriculation), sex, UIM status, non-US citizen, and age at college graduation. For post-program outcomes the input metrics included applicant metrics listed above and the in-program metric, TTD.

The second dataset included all students who graduated or withdrew from the Albert Einstein College of Medicine MSTP from 2006 to 2022 and from the University of Pennsylvania Perelman School of Medicine MSTP from 2007 to 2024. The Penn-Einstein dataset includes metrics from the national dataset plus the normalized admissions committee score for each individual, attrition, the number of first (does not include co–first author papers where the student was not the first author) and total publications resulting from their research at the respective institution, and the average and total impact factor of the journals in which those publications appeared. The journal impact factors were obtained from 2022 Web of Science data (accessed October 24, 2023). There were 593 individuals (Einstein, *N* = 257; Penn, *N* = 336). Of these 255 had completed postgraduate training (Einstein, *N* = 138 in 2006–2018; Penn, *N* = 117 in 2007–2018).

The output metrics for the machine learning model required definition of favorable and unfavorable outcomes as defined in Table 1. For the multivariable linear regression analysis of the Penn-Einstein dataset, the outcome was prediction of the number of PhD publications and impact factors based on applicant metrics.

### Machine learning analysis.

Analyses were conducted in Python (3.9) using the Scikit-learn package for machine learning. Logistic regression was used as the classifier algorithm to predict the binary outcome variable of interest. In each case, 70% of the data was used for training purposes and the remaining 30% for testing; 20 replicates of each training and testing session were used to generate the 95% confidence intervals (Figure 1). The random forest algorithm was also used for classification prediction with similar results. General guidelines suggest that a value of 0.70 for the AUC_ROC is the lower threshold of acceptable discrimination (49).

### Statistics.

To compute statistical differences between groups (Figure 2 and Supplemental Figures 1–3, 5, and 6), we used the 2-sided nonparametric Mann-Whitney test with asymptotic *P* value calculation given the large sample size. *P* < 0.05 was considered statistically significant.

### Study approval.

This work was performed under the auspices of Albert Einstein College of Medicine IRB Protocol 2021-12869. It was deemed exempt from federal human research regulations (45-CFR-46).

### Data availability.

Data from the National MD-PhD Program Outcomes Study are available by request to AAMC (https://www.aamc.org/request-aamc-data). A list of data elements to request is included in the Supporting Data Values file associated with this paper.

## Author contributions

MHA and LFB conceived the project. MHA, LFB, and MT designed the study. MHA, LFB, MT, and AW acquired data. MT, LFB, and MHA analyzed data. MHA, LFB, MT, and AW wrote the manuscript. All the authors accept responsibility for its contents. LFB and MHA have alternated first versus last author on numerous papers resulting from their collaborative efforts. MT made major contributions to the project and data analysis and thus is second author and co–corresponding author. AW assisted in collecting University of Pennsylvania data and is third author.

## Supplementary Material

Supplemental data

Supporting data values

## Figures and Tables

**Table 1:**
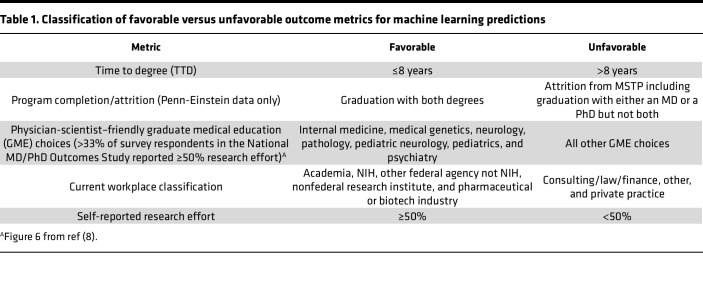
Classification of favorable versus unfavorable outcome metrics for machine learning predictions

## Version 1 03/04/2025

In-Press Preview

## Version 2 04/08/2025

Electronic publication
