# Supplementary material for: Admissions to MD-PhD programs: how well do application metrics predict short- or long-term physician-scientist outcomes?
Source: JCI Insight. 2025 Mar 4;10(7):e184493. doi: 10.1172/jci.insight.184493 (PMC11981614; doi:10.1172/jci.insight.184493)
Supplement: Supplemental data [file jciinsight-10-184493-s017.pdf]

Supplementary Materials

Admissions to MD-PhD programs: How well do application metrics predict short or long-term physician-scientist outcomes?

Lawrence F. Brass[1], Maurizio Tomaiuolo[2], Aislinn Wallace[3], Myles H. Akabas[4]

[1]Lawrence F. Brass
Departments of Medicine and Pharmacology
University of Pennsylvania Perelman School of Medicine
Philadelphia, Pennsylvania, USA
brass@pennmedicine.upenn.edu

[2]Maurizio Tomaiuolo
Department of Pediatrics
Children's Hospital of Philadelphia
Philadelphia, Pennsylvania, USA
tomaiuolom@chop.edu

[3]Aislinn Wallace
Office of Biomedical Graduate Studies
University of Pennsylvania Perelman School of Medicine
Philadelphia, Pennsylvania, USA

[4]Myles H. Akabas
Departments of Neuroscience and Medicine
Albert Einstein College of Medicine
1300 Morris Park Avenue
Bronx, New York, USA
myles.akabas@einsteinmed.edu

Address correspondence to MHA, LFB, and MT.

LFB, MT, and MHA contributed equally.

[3]Aislinn Wallace
Current address:
Department of Criminal Justice and Criminology
Georgia State University
P.O. Box 3992
Atlanta, Georgia 30302-3992
awallace48@gsu.edu

Supplementary Materials

**Table of Contents**	**Page number**
**Figure S1.** Histograms of applicant metrics grouped by sex	3
**Figure S2.** Histograms of applicant metrics grouped by whether the individual belongs to a group underrepresented in medicine (UiM) by NIH definitions.	4
**Figure S3.** Histograms of applicant metrics grouped by high reported research effort (RE high) vs low reported research effort (RE low).	5
**Figure S4.** Machine learning prediction of TTD and choice to train in a research friendly clinical specialty is not improved by limiting the analysis to the subset of graduates who graduated in or after 1995 (N=3,401).	6
**Figure S5.** Histograms and mean values of the numbers of first author and total PhD publications and the average and total impact factors of the journals in which the papers were published for graduates of the Penn (n=117) and Einstein (n=138) MSTPs.	7
**Figure S6.** Histograms of uGPA and MCAT for the Penn and Einstein MSTPs.	8
**Figure S7.** Applicant metrics do not predict average or total impact factor of journals in which PhD papers were published or the total number of publications for the Penn-Einstein data set.	9
**Figure S8.** Machine learning analysis using only the highest and lowest quintiles for the indicated applicant parameter does not predict current workplace.	10
**Figure S9.** Machine learning analysis using only individuals in the highest and lowest quintiles for the indicated applicant parameter does not predict research effort.	11
**Table S1.** Summary of input metrics, outputs, and methods for comparisons for the analysis done in this work.	12
**Table S2**. Number of missing values for each National Outcomes data set variable, the percent of the total, and the method of imputation.	14
**METHODS**	15

Supplementary Materials

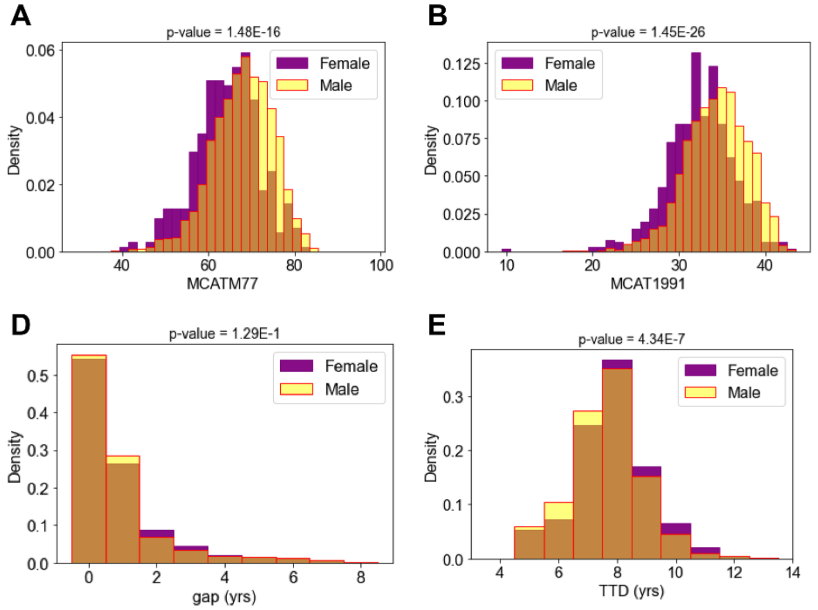

F

Variable	Mean Females	Mean Males	N
MCATM77	64.25	67.84	1730
MCAT1991	32.40	34.26	2203
GPA	3.70	3.68	4659
gap	1.01	0.87	3892
TTD	7.86	7.64	4659

Figure S1. Histograms of applicant metrics subdivided by sex. Women are in purple, men in yellow, and the overlap is shown in brown. P-values above each panel indicates the significance of the difference between men and women. Distributions of **(A)** MCATM77 scores, **(B)** MCAT1991 scores, **(C)** undergraduate GPA, **(D)** length of gap between undergraduate college graduation year and MD-PhD matriculation year, and **(E)** time to degree (TTD) calculated as year of MD-PhD graduation minus year of matriculation. **(F)** Mean values for women and men, and number of individuals for each variable

Supplementary Materials

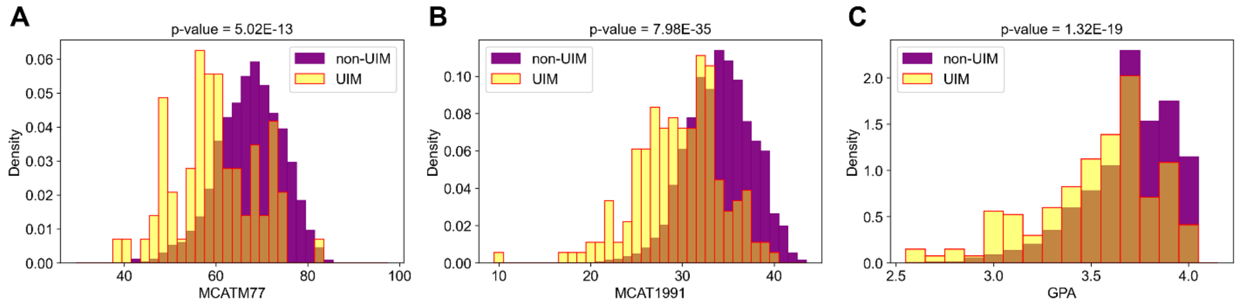

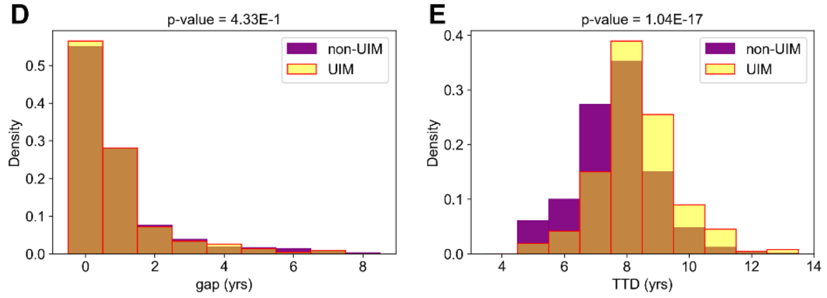

F

Variable	Mean non-UIM	Mean UIM	N
MCATM77	67.43	59.74	1730
MCAT1991	34.07	29.62	2203
GPA	3.70	3.53	4659
gap	0.92	0.77	3892
TTD	7.65	8.33	4659

Figure S2. Histograms of applicant metrics subdivided by whether the individual belongs to a group underrepresented in medicine (UIM) by NIH definitions. UIM are shown in yellow, non-UIM are in purple, and the overlap between the two groups is shown in brown. P-values above each panel indicates the significance of the difference between UIM and non-UIM groups. Distributions of **(A)** MCATM77 scores, **(B)** MCAT1991 scores, **(C)** undergraduate GPA, **(D)** length of gap between undergraduate college graduation year and MD-PhD matriculation year, and **(E)** time to degree calculated by year of MD-PhD graduation minus year of matriculation. **(F)** Mean values for non-UIM and UIM groups, and number of individuals for each variable.

Supplementary Materials

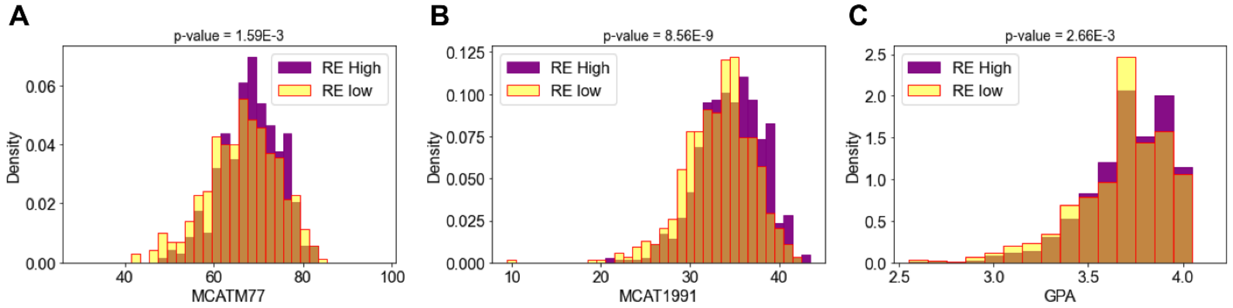

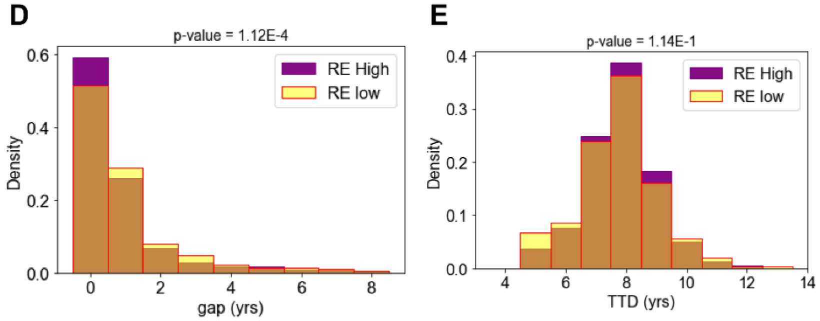

F

Variable	Mean RE High	Mean RE Low	N
MCATM77	68.22	62.32	695
MCAT1991	34.53	33.20	2187
GPA	3.71	3.68	2187
gap	0.81	1.09	1846
TTD	7.83	7.78	2187

Figure S3. Histograms of applicant metrics subdivided by those who reported high research effort (RE high) vs those who reported low research effort (RE low). RE low is shown in yellow, RE high is in purple, and the overlap is shown in brown. P-values above each panel indicates the significance of the difference between men and women. Distributions of **(A)** MCATM77 scores, **(B)** MCAT1991 scores, **(C)** undergraduate GPA, **(D)** length of the gap between college graduation year and MD-PhD matriculation year, and **(E)** time to degree (TTD) calculated by year of MD-PhD graduation minus year of matriculation. **(F)** Mean values of high and low research effort groups, and number of individuals for each variable.

Supplementary Materials

Sub-cohort of alumni with graduation ≥ 1995 (N=3401)

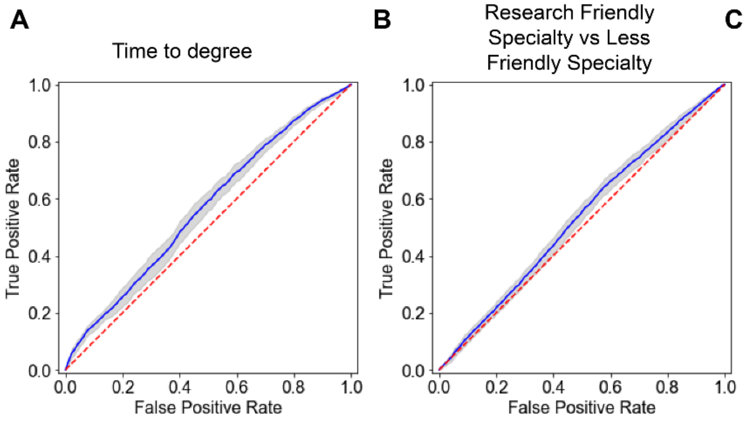

C

Machine Learning Setup		
Data Set	**(A)** National outcomes graduated on or after 1995	**(B)** National outcomes graduated on or after 1995
Outcome	Time to degree	Research friendly specialty
Favorable	≤ 8 (N=2,107; 70%)	research oriented (N=1,820; 60%)
Unfavorable	>8 (N=934; 30%)	less research oriented (N=1,221; 40%)
Features	Age at college graduation	Age at college graduation
	GPA	GPA
	MCAT	MCAT
	Gap years	Gap years
	UIM	UIM
	US citizenship	US citizenship
	Sex	Sex
		Time to degree
Classifier	Logistic regression	
**Classifier results**		
Precision	0.71	0.61
Recall	0.52	0.54
F1	0.60	0.58
Accuracy	0.53	0.53
ROC_AUC	0.56	0.53

Figure S4. Machine learning prediction of TTD and choice to train in a research friendly clinical specialty is not improved by limiting the analysis to the subset of graduates who graduated in or after 1995 (N=3,401). This analysis was performed to determine whether increasing average TTD or changing choices of GME specialty were limiting the ability of the machine learning analysis to predict these outcomes. **(A)** Receiver operating characteristic curve for machine learning analysis of the relationship between the input metrics and the time to degree outcome. The input metrics used in the analysis are listed under Features in Panel C. The outcome metric was time to degree characterized as favorable (TTD≤8 years) and unfavorable (TTD>8 years) (Table 1). **(B)** Outcome as choice of research friendly vs less friendly medical specialties. Receiver operating characteristic curve for machine learning analysis of the relationship between the input features and medical specialty choice. The outcome metric was medical specialty choice characterized as favorable (medical specialties with more than 33% of alumni reporting ≥50% research effort) and unfavorable (all other specialties) (Table 1). **(C)** The input features, outcome metrics, and classifier results for the analysis in Panels A and B.

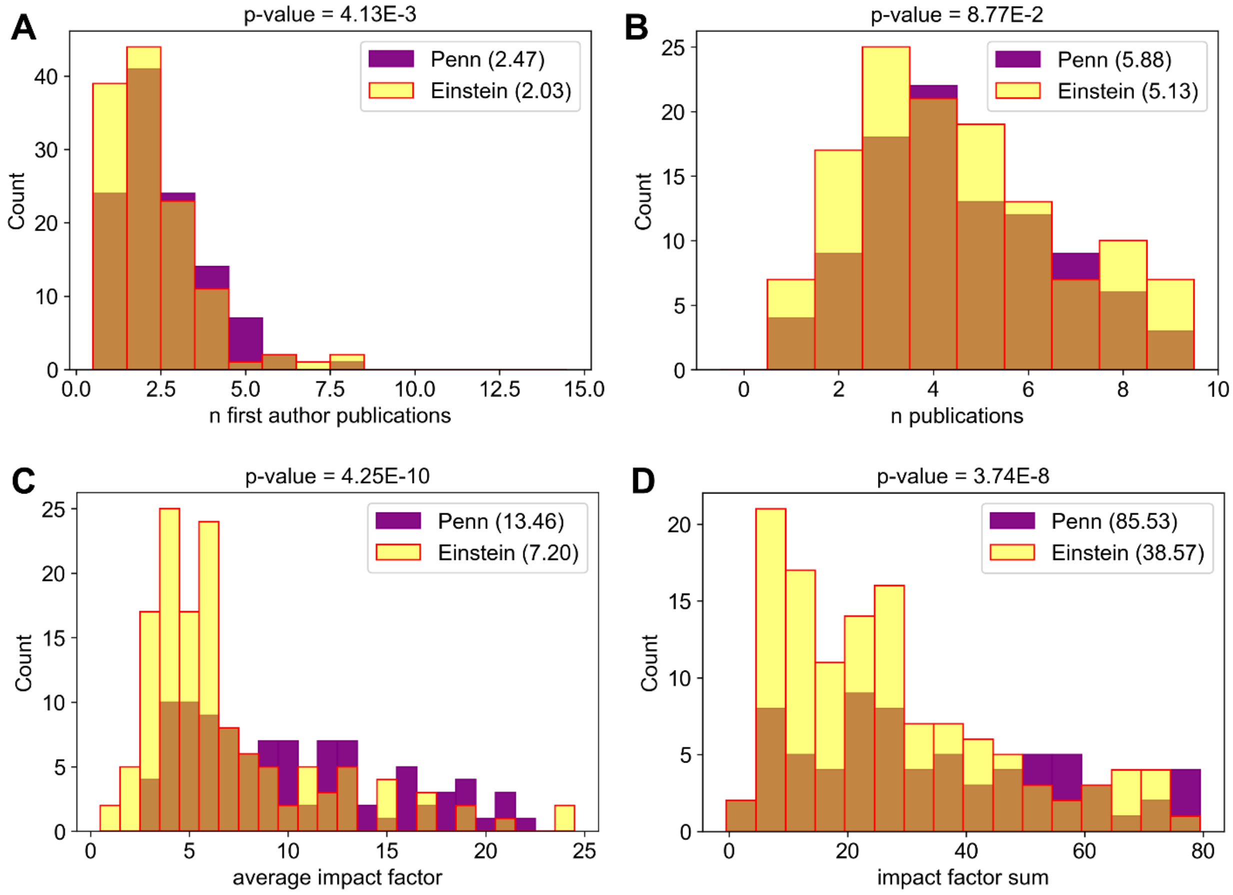

Figure S5. Publication numbers and journal impact factors for graduates of the Penn (n=117) and Einstein (n=138) MSTPs. Penn data are in purple, Einstein data are in yellow. Brown represents the overlap of the histograms. Mean values are in parenthesis in each key. **(A)** Number of first author papers from PhD thesis research. **(B)** Total number of papers from PhD thesis research. **(C)** Average impact factor of the journals in which the papers were published. **(D)** Sum of the impact factors of the PhD papers. P-values comparing the distributions are shown above each panel.

Supplementary Materials

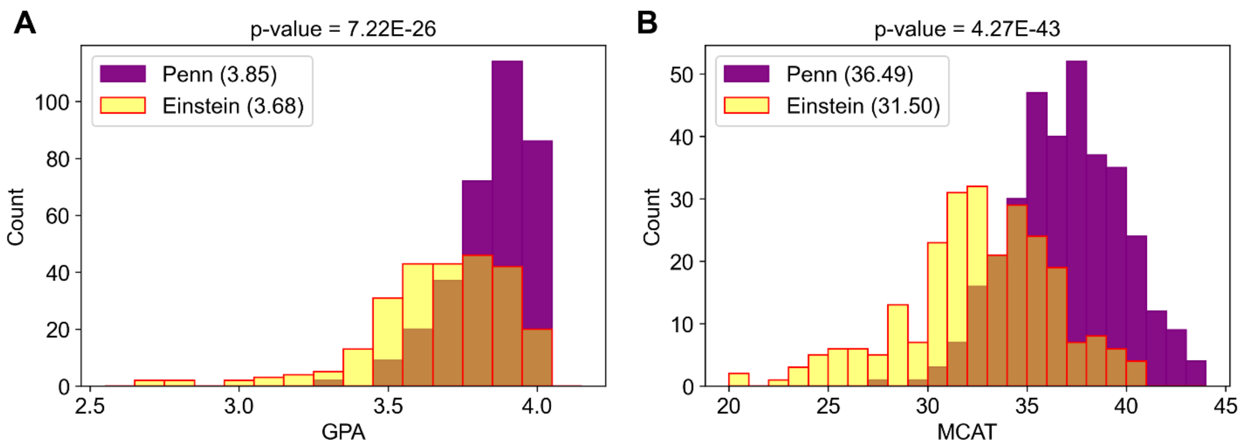

Figure S6. Histograms of GPA (**A**) and MCAT (**B**) for the Penn and Einstein MSTPs. P-values comparing the distributions are shown above each panel. (Total, N=593; Einstein, N=257; U Penn, N=336).

Supplementary Materials

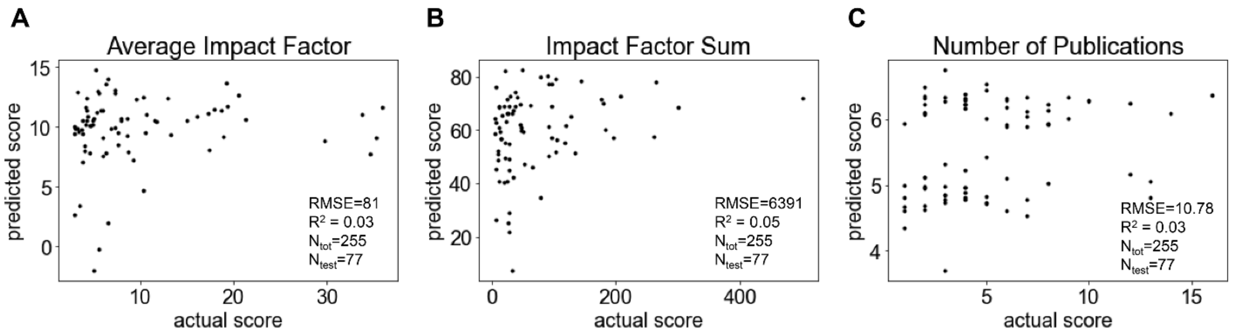

Figure S7. Applicant metrics do not predict average or total impact factor of journals in which PhD papers were published or the total number of publications for the Penn-Einstein data set. Scatter plots of actual vs multivariable linear regression predicted values for **(A)** average impact factor of journals in which PhD papers were published, **(B)** total impact factor of journals in which PhD papers were published, and **(C)** total number of PhD papers. All applicant metrics for the Penn-Einstein data set were used in the analysis. Applicant metrics include age at college graduation, GPA, MCAT, gap length, admissions committee summative score, UIM, citizenship, and sex.

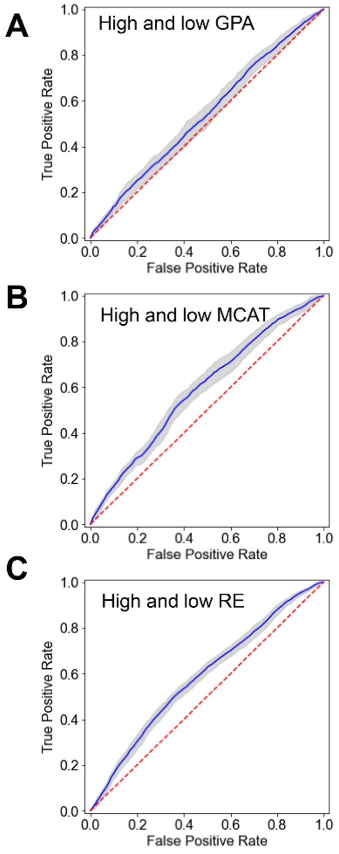

D

Machine Learning Setup			
Data Set	**(A)** National outcomes upper and lower GPA quintiles (N=1,949)	**(B)** National outcomes upper and lower MCAT quintiles (N=2,034)	**(C)** National outcomes upper and lower Research Effort quintiles (N=2,187)
Outcome	Favorable workplace		
Favorable	Academia, Government, Pharma/Biotech, Research Institute (N=1,540; 79%)	Academia, Government, Pharma/Biotech, Research Institute (N=1,647; 80%)	Academia, Government, Pharma/Biotech, Research Institute (N=1,687; 78%)
Unfavorable	All other (N=409; 21%)	All other (N=387; 20%)	All other (N=500; 22%)
Features	Age at college graduation<br>GPA<br>MCAT<br>Gap years<br>UIM<br>US citizenship<br>Sex<br>Time to degree		
Classifier	Logistic regression		
**Classifier results**			
Precision	0.21	0.61	0.29
Recall	0.43	0.50	0.56
F1	0.29	0.32	0.40
Accuracy	0.56	0.61	0.56
ROC_AUC	0.53	0.59	0.58

Figure S8. Machine learning analysis using only individuals in the highest and lowest quintiles of **(A)** college GPA, **(B)** MCAT, and **(C)** research effort does not improve predictions of a favorable current workplace. The input metrics used in the analysis are listed under Features in Panel D. The receiver operating characteristic area under the curve (ROC_AUC) would be 0.0 if the model predictions were completely wrong, 1.0 if the predictions were completely correct, and 0.5 if the predictions were random (red dashed line). The blue line shows the calculated curve. The gray lines show the 95% confidence interval for the repeated trials of the model. **(D)** The values of commonly used prediction metrics are shown in the 'Classifier results' table. A value of 1 for each metric would indicate all correct predictions.

Supplementary Materials

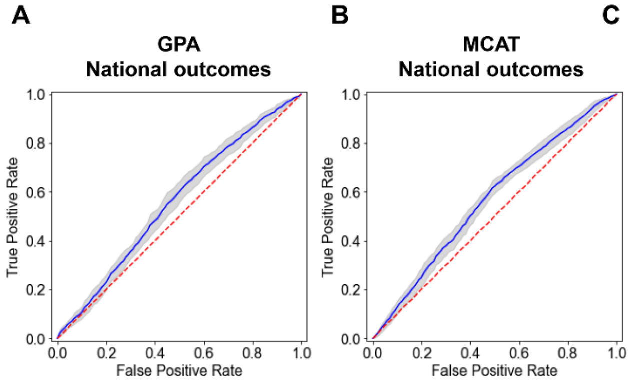

C

Machine Learning Setup		
Data Set	National outcomes in	
Partition	**(A)** GPA top 80% (>=3.9) and bottom 20% (<=3.5) (N_{fav}=763, 50%; N_{unf}=739, 50%)	**(B)** MCAT top 80% (>=84) and bottom 20% (<=65) (N_{fav}=847, 51%; N_{unf}=796, 49%)
Outcome	Research effort	
Favorable	≥50%	
Unfavorable	<50%	
Features	Age at college graduation	Age at college graduation
		GPA
	MCAT	
	Gap years<br>UIM<br>US citizenship<br>Sex<br>Time to degree	
Classifier	Logistic regression	
**Classifier results**		
Precision	0.53	0.54
Recall	0.60	0.56
F1	0.57	0.56
Accuracy	0.55	0.55
ROC_AUC	0.57	0.57

Figure S9. Machine learning analysis using only individuals in the highest and lowest quintiles of **(A)** college GPA and **(B)** MCAT does not improve predictions of research effort. **(C)** The input metrics used in the analysis are listed under Features. The outcome metric was self-reported research effort dichotomized as favorable ≥ 50% and unfavorable <50% (Table 1). The receiver operating characteristic area under the curve (ROC_AUC) would be 0.0 if the model predictions were completely wrong. ROC_AUC would be 1.0 if the predictions were completely correct, and 0.5 if the predictions were random (red dashed line). Blue line shows calculated curve, gray shows the 95% confidence interval for the repeated trials of the model. The values of commonly used prediction metrics are shown in the 'Classifier results' table in Panel C. A value of 1 for each metric would indicate all correct predictions.

Supplementary Materials

Table S1. Summary of input metrics, outputs, and methods for comparisons for the analysis done in this work.

Dataset	Inputs	Method of comparison	Outputs	Figure
National	MCAT, GPA, gap length, time to degree	Histogram	Favorable Vs. unfavorable workplace	2A-F
National	MCAT, GPA, time to degree, gap length	Spearman's correlation	Pairwise correlation coefficients	2G-L
National	Age at college graduation, GPA, MCAT, gap length, UIM, citizenship, sex	Machine learning	Time to degree	3A
Penn-Einstein	Age at college graduation, GPA, MCAT, gap length, UIM, citizenship, sex, summative scores	Machine learning	Attrition from MD-PhD program	3B
National	Age at college graduation, GPA, MCAT, gap length, UIM, citizenship, sex	Machine learning	Medical specialty	3C
National	Age at college graduation, GPA, MCAT, gap length, UIM, citizenship, sex, time to degree	Machine learning	Current workplace	4A
National	Age at college graduation, GPA, MCAT, gap length, UIM, citizenship, sex, time to degree	Machine learning	Research effort	4B
Penn-Einstein	Age at college graduation, GPA, MCAT, gap length, UIM, citizenship, sex, summative scores, publications	Machine learning	Current workplace	4C
National	MCAT, GPA, gap length, time to degree	Histogram	Comparison of women and men	S1
National	MCAT, GPA, gap length, time to degree	Histogram	UIM status	S2
National	MCAT, GPA, gap length, time to degree	Histogram	Research effort	S3
National (limited to graduates after 1994)	Age at college graduation, GPA, MCAT, gap length, UIM, citizenship, sex	Machine learning	Time to degree	S4A
National (limited to graduates after 1994)	Age at college graduation, GPA, MCAT, gap length, UIM, citizenship, time to degree, sex	Machine learning	Research effort	S4B
Penn-Einstein	Publication number and impact	Histogram	Comparison of Penn and Einstein	S5

Supplementary Materials

Dataset	Inputs	Method of comparison	Outputs	Figure
Penn-Einstein	GPA and MCAT	Histogram	Comparison of Penn and Einstein	S6
Penn-Einstein	Age at college graduation, GPA, MCAT, gap length, UIM, citizenship, sex, summative scores, publications	Multivariable linear regression	Publication number and impact	S7
National	Age at college graduation, GPA, MCAT, gap length, UIM, citizenship, time to degree, sex (highest and lowest GPA)	Machine learning	Current workplace	S8A
National	Age at college graduation, GPA, MCAT, gap length, UIM, citizenship, time to degree, sex (highest and lowest MCAT)	Machine learning	Current workplace	S8B
National	Age at college graduation, GPA, MCAT, gap length, UIM, citizenship, time to degree, sex (highest and lowest research effort)	Machine learning	Current workplace	S8C
National	Age at college graduation, GPA, MCAT, gap length, UIM, citizenship, time to degree, sex (highest and lowest MCAT)	Machine learning	Research effort	S9A
National	Age at college graduation, GPA, MCAT, gap length, UIM, citizenship, time to degree, sex (highest and lowest GPA)	Machine learning	Research effort	S9B

Supplementary Materials

Feature	N tot	N missing	% missing	Imputation method	Variance* pre-imputation scaled by population size	Variance* post-imputation scaled by population size	% scaled variance decrease
AGE_AT_UG_GRAD	4659	401	8.6%	mean	0.00026	0.00022	16.5
gap	4659	767	16.5%	assume yes	0.00006	0.00006	11.3
TTD (yrs)	4659	102	2.2%	mean	0.00036	0.00032	11.3
GPA	4659	500	10.7%	mean	0.00002	0.00001	20.3
MCAT	4659	726	15.6%	mean	0.01938	0.01794	7.4
SEX	4659	5	0.1%	frequency based	not computed		
URM	4659	0	0.0%				
non-US citizen	4659	0	0.0%				

*Variance scaled by population size = Var (x) / N (x)

Table S2. Number of missing values for each National Outcomes data set variable, the percent of the total, and the method of imputation. Of the 4,659 respondents, 3,754 (80.6%) had values for all variables, and 905 (19.4%) were missing one or more values. Of those 905 missing values, at least 483 (53%) graduated prior to 1985, and an additional 61 (7%) lacked a graduation date but had matriculation dates prior to 1975. Another 32 (3.5%) lacked both matriculation and graduation dates. 329 (36%) had graduation dates between 1985 and 2014 (113 between 1985-94; 170 between 1995-04; 46 between 2005-14).

Supplementary Materials

METHODS

Data sources. Two datasets were used for this study. One dataset was derived from the 2015 National MD-PhD Program Outcomes Study and is referred to as national outcomes dataset (1-4). It included survey data from 4,659 graduates of 80 MD-PhD programs who matriculated between 1965 and 2007 and who had completed postgraduate training when they completed the survey in 2015. Respondents characterized their current position (academia full time, academia part time, consulting/law/finance, Federal agency other than NIH, NIH, other, pharmaceutical/biotechnology industry, private practice, research institute), allocated percent of professional effort devoted to research, clinical care, teaching, administration, other; types of funding they had or previously had received, graduate medical education (GME) specialty in which they had trained. The survey responses were merged with person-level information from the AAMC database that among other information including sex, race/ethnicity, age at college graduation, year of college graduation, and year of MD-PhD program matriculation and graduation. For the current work, the AAMC added undergraduate GPA at the time of AMCAS application submission, and the most recent total MCAT score for each individual survey respondent. The MCAT exam changed structure and scoring for individuals included in the national dataset in 1991. From 1977 to 1991 the MCAT exam was scored on a scale from 6 to 90. From 1991 to 2015 the MCAT was scored on a 3 to 45 scale. Based on race/ethnicity data, we defined groups underrepresented in medicine (UIM) to include those who reported identifying as Black or African American, Hispanic or Latino, American Indian or Alaska Native, or Native Hawaiian or Other Pacific Islander. Non-UIM included all individuals who did not identify as belonging to one of those groups. The non-UIM group includes 187 non-US citizens and 110 individuals for whom race/ethnicity was reported as unknown or other (1-3). The de-identified person-level data was provided to the authors by AAMC in an Excel file. The following data elements were used in this analysis: age at college graduation, undergraduate GPA, MCAT score, number of gap years between college graduation and MD-PhD program

matriculation, underrepresented in medicine status (yes or no), US citizenship (yes or no), sex, time to completion of both degrees (TTD = year of graduation minus year of matriculation), self-reported percent research effort, and category of current place of employment.

The second dataset included all students who graduated or withdrew from the Albert Einstein College of Medicine MSTP from 2006-2022 and from the University of Pennsylvania Perelman School of Medicine MSTP from 2007 to 2024. The Penn-Einstein dataset includes data as in the National MD-PhD Program Outcomes Study plus the admissions committee scores for each individual, attrition, the number of first and coauthored publications resulting from their research at the respective institution, and the average and total journal impact factor of the journals in which those publications appeared. There were 593 individuals (Einstein, N=257; U Penn, N=336). Of these 255 had completed postgraduate training (Einstein, N=138 (2006-18); U Penn, N=117 (2007-18).

The machine learning analysis of the national dataset was divided into two endpoints, prediction of in-program and post-program outcomes. For in-program outcomes the input features used were applicant metrics that included: final MCAT score, undergraduate GPA reported in the AMCAS application, gap length (# of years between college graduation and MD-PhD program matriculation), sex, UIM status, non-US citizen, and age at college graduation. For post-program outcomes the input metrics included applicant metrics as defined above, and in-program metrics, time to degree (TTD).

For the Penn-Einstein dataset, additional features included the normalized admissions committee score, number of publications from PhD thesis research including total and first author (does not include co-first author papers where the student was not the first author), and

impact factor of the publications, both average, and sum of the paper impact factors. The impact factors for the journals were obtained from 2022 Web of Science data (accessed 10/24/2023).

The output metrics for the machine learning model required a definition of favorable and unfavorable outcomes. These are summarized in Table 1. For the Penn-Einstein dataset, additional outcome metric was attrition from the program. For the multivariable linear regression analysis of the Penn-Einstein dataset, the outcome was prediction of the number of PhD publications (first and co-author) and impact factors based on applicant metrics.

Imputed data. Missing data for the Penn, Einstein, and the national datasets were handled through imputation. Missing values for MCAT, GPA, and age at college graduation were assigned the mean of the observed values. Mean imputation was also used for time to degree when either the actual was missing, or the reported TTD was 4 years or less. Missing values for UIM status and US citizenship were assigned the default values of no and yes respectively. Missing values for the number of gap years were assigned the most common value (i.e. 1 year). Finally, in order to use the values from the two different MCAT test scores (MCAT1991 and MCATM77) in a single analysis both scores were normalized dividing by their respective maximum value and multiplied by 100. Percent of missing data in the National Outcomes dataset for each input feature, the method of imputation, and the corresponding reduction in rescaled variance are shown in Table S2. Of the 4,659 respondents, 3,754 (80.6%) had all values for all variables, and 905 (19.4%) were missing one or more values. Of those 905 missing values, at least 483 (53%) graduated prior to 1985, and an additional 61 (7%) lacked a graduation date but had matriculation dates prior to 1975. Another 32 (3.5%) lacked both matriculation and graduation dates. 329 (36%) had graduation dates between 1985 and 2014 (113 between 1985-94; 170 between 1995-04; 46 between 2005-14). Out of 4,659 respondents, 273 lacked either a year of matriculation or year of graduation (N=102) or had a TTD <5 years

(N=170) where we assumed there was a mistake in one of the dates, or a TTD >16 years (N=1) where we likewise assumed a mistake. So 5.9% of the survey respondents lacked a TTD and this was imputed to be 8 years.

Machine learning analysis. Analyses were conducted in Python (3.9) using the Scikit-learn package for machine learning. Logistic regression was used as the classifier algorithm to predict the binary outcome variable of interest. In each case, 70% of the data was used for training purposes and the remaining 30% for testing; 20 replicates of each training and testing session were used to generate the 95% confidence intervals (Figure 1). The 'random forest' algorithm was also used for classification prediction with similar results. General guidelines suggest that a value of 0.70 for the area under the curve of the Receiver Operating Characteristic (AUC_ROC) is the lower threshold of acceptable discrimination (5).

REFERENCES

1. Akabas MH, Tartakovsky I, and Brass LF. National MD-PhD Program Outcomes Study. Association of American Medical Colleges. Washington, DC. 2018 https://store.aamc.org/national-md-phd-program-outcomes-study.html Accessed 4/11/2024.
2. Brass LF, and Akabas MH. The national MD-PhD program outcomes study: Relationships between medical specialty, training duration, research effort, and career paths. *JCI Insight.* 2019;4(19):e133009.
3. Akabas MH, and Brass LF. The national MD-PhD program outcomes study: Outcomes variation by sex, race, and ethnicity. *JCI Insight.* 2019;4(19):e133010.
4. Andriole DA, Grbic D, Yellin J, and McKinney R. MD-PhD Program Graduates' Engagement in Research: Results of a National Study. *Acad Med.* 2021;96(4):540-8.
5. Hosmer Jr. DW, Lemeshow S, and Sturdivant RX. *Applied Logistic Regression.*Third Edition. John Wiley & Sons, Inc.; 2013.
